# Cost-Effectiveness of Four Tobacco Control Interventions in Mongolia

**DOI:** 10.1093/ntr/ntad111

**Published:** 2023-07-21

**Authors:** Ariuntuya Tuvdendorj, Talitha Feenstra, Erik Buskens

**Affiliations:** Department of Health Policy, School of Public Health, Mongolian National University of Medical Sciences, Ulaanbaatar, Mongolia; Department of Epidemiology, University Medical Center Groningen, University of Groningen, Groningen, The Netherlands; Department of Epidemiology, University Medical Center Groningen, University of Groningen, Groningen, The Netherlands; Faculty of Science and Engineering, Groningen Research Institute of Pharmacy, University of Groningen, Groningen, The Netherlands; Faculty of Science and Engineering, Centre for Nutrition, Prevention, and Health Services, Institute for Public Health and the Environment, Bilthoven, The Netherlands; Department of Epidemiology, University Medical Center Groningen, University of Groningen, Groningen, The Netherlands

## Abstract

**Introduction:**

The aim of this study is to quantify the cost-effectiveness of four tobacco control interventions: Tobacco taxation, mass media campaigns, school programs, and cessation support, and to illustrate how available evaluation tools can be adapted to the local setting.

**Aims and Methods:**

We used the dynamic population health modeling-health impact assessment tool to project the future smoking prevalence associated with the interventions and to simulate the resulting smoking-related disease burden over time. Applying the most recent available national Mongolian data as input, the costs and effects of four interventions were compared to a business-as-usual scenario, resulting in costs per life year gained and per disability-adjusted life years (DALYs) averted.

**Results:**

Three years after implementation, all interventions reduce the prevalence of current smoking, with the strongest reduction observed with the increase in tobacco tax (5.1% points), followed by mass media campaigns (1.6% points), school programs (1.3% points), and cessation support interventions (0.6% points). School programs were a cost-saving tobacco control intervention compared to current practice in Mongolia, while the other programs resulted in additional costs compared to business as usual. Compared to the World Health Organization (WHO) thresholds, all interventions would be considered “very cost-effective” in terms of cost per DALY averted (below US$ 4295 per DALY averted) in Mongolia.

**Conclusions:**

Large-scale interventions such as taxation and mass media campaigns result in both cost-effectiveness and important health benefits in relation to intervention costs. Reducing the prevalence of smoking among the male population would be particularly worthwhile in Mongolia.

**Implications:**

This study shows that in Mongolia school programs were a cost-saving intervention, while the cost-effectiveness ratios were US$ 25 per disability-adjusted life year (DALY) averted for mass media campaigns, US$ 74 for taxation, and US$ 1961 for cessation support interventions. Compared to the WHO thresholds, all interventions would be considered “very cost-effective” in terms of expenses per DALY averted (<US$ 4295 per DALY averted), making it beneficial to scale up the WHO-Monitor tobacco use and prevention, Protect people from smoke, Offer help to quit smoking, Warn about the danger, Enforce bans, and Raise taxes (MPOWER) interventions to reduce the burden from smoking in Mongolia.

## Introduction

A range of interventions have been proposed to reduce tobacco use. The World Health Organization (WHO) introduced an Monitor tobacco use and prevention, Protect people from smoke, Offer help to quit smoking, Warn about the danger, Enforce bans, and Raise taxes (MPOWER) package of six cost-effective measures: Monitor tobacco use and prevention, Protect people from smoke, Offer help to quit smoking, Warn about the danger, Enforce bans, and Raise taxes.^1^ Countries are encouraged to adopt at least one measure at best-practice level—for example, complete smoke-free laws covering indoor public places, workplaces and public transport, large graphic pack warnings, or levying excise taxes of at least 75% of the retail prices. The WHO has set the global target of reaching what they call a tobacco endgame—a world where less than 5% of the adult population use tobacco by 2040. In Asia, smoking prevalence is currently still very high, with levels of approximately 27% in the adult population.^2^

In 2013, the Mongolian government launched a national tobacco control strategy, which sets specific targets for 2021 for a reduction in smoking among adolescents (age 13–15) from 5.9% to 4.9%, and among adults from 27.1% to 21.7%.^3^ In line with national and international initiatives, a range of tobacco control measures have been adopted. A health promotion fund was launched in 2012, funded by 1% of tobacco tax revenues, 1% of alcohol tax revenues, and 2% of tax revenues from the sale of medicines. These funds have been used to implement a range of health promotion interventions, including tobacco control through mass media campaigns and educational programs.

To achieve maximal return on investment, policy makers are challenged to prioritize key interventions for tobacco control based on their cost-effectiveness in local healthcare systems.^4^ Evidence-based decision-making has so far been limited, especially in resource-limited settings. Among the few studies that were conducted in low to middle-income countries, the costs per disability-adjusted life year (DALY) gained varied from $3 to $70 for a 10% tax rise, and from $280 to $870 for smoking cessation.^5^ Most of these studies made use of local cost information, and local data on smoking prevalence, but they did not apply local public health models, or representative relative risks. Hence, cost-effectiveness for the different policy options has varied greatly, and robust estimates over the complete range of interventions are still lacking.

One-way forward is to use decision analytic models as part of a data synthesis approach to offer estimates of the long-term costs and benefits of health interventions.^6^ Various modeling tools and costing templates are available to support local cost-effectiveness studies in support of public health policy.^7^ However, there is still considerable heterogeneity regarding input parameters and model structures. Especially in Asia, most model-based economic evaluations of tobacco control interventions lack relevant local input data and apply a static model structure.^8^ To allow comparable estimates for a range of interventions, dynamic health impact assessment models are better suited as model structures, since they make it possible to include benefits from reduced morbidity as well as mortality, and they provide more realistic insights than static models into the delay of health benefits, the potential waning of effects over time, and the timing of savings from reduced disease burden. Several public health modeling frames are available that support the development of a dynamic health impact assessment model for tobacco control. These have been applied in various settings, but they require careful integration of local input data.^9^ Cost information must also be integrated to allow for an evaluation of the cost-effectiveness of the interventions.

The aim of the current study is to inform policy makers about the cost-effectiveness of four population-based tobacco control interventions in Mongolia. All interventions were based on the WHO-MPOWER package, except the school-based program. This was included as an important intervention to support prevention of smoking among youth. As such, the study serves as an example of how existing modeling tools can be combined with local data to produce tailored evidence for local policy decision-making.

## Methods

**Figure 1.**
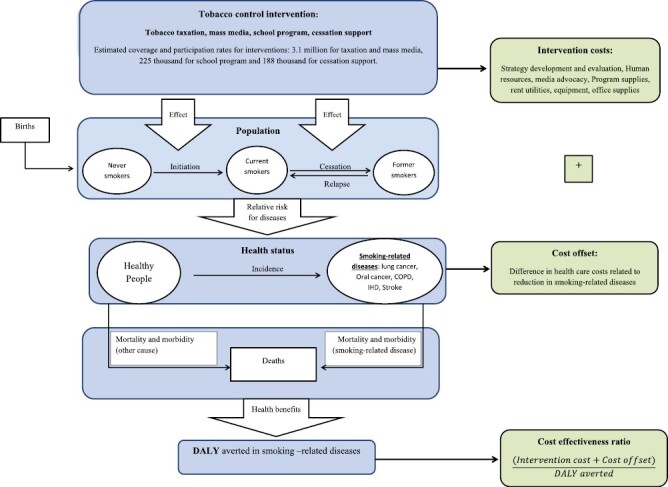
Conceptual framework, and outcomes.

We used the dynamic population health modeling-health impact assessment (DYNAMO-HIA) tool to project the future smoking prevalence associated with four tobacco control interventions, and to simulate the resulting smoking-related disease burden over time.^10^ This was then combined with cost information to calculate costs per DALY averted and costs per life year gained (LYG).^11^ The conceptual framework and outcomes are summarized in Figure 1.

The introduction of tobacco control interventions has five consecutive consequences: Firstly, it imposes intervention costs related to the implementation and enforcement of each intervention; secondly, it affects smoking initiation and smoking cessation rates in the age groups targeted by the intervention; thirdly, it reduces smoking prevalence and may increase the prevalence of former smokers because of changes in smoking behavior; fourthly, it reduces smoking-related diseases as a consequence of a reduction in smoking prevalence; finally, it saves future costs related to reduced health care needs for smoking-related diseases (cost-offsets), and may lead to higher costs of care for diseases associated with old age, as people live longer. Finally, it results in life years gained and improved quality of life, expressed as DALYs averted because of the reduction in smoking-related diseases. DALYs combine losses in quality and quantity of life, so DALYs averted reflect gains in terms of morbidity and mortality.^11^

Overall, the model requires four sets of input parameters; demographic data, smoking prevalence data, cost data, and epidemiological data, stratified by sex and age in 1-year age categories up to the age 95.^12^ Country-specific input sources were used where possible. The input parameters and their sources are described in detail in Supplementary Table S1. Data on population size, overall mortality, and forecasted numbers of births were collected from the Mongolian national statistical information service in 2019.^13^Supplementary Table S2 presents observed age-group-specific and gender-specific smoking prevalence data.

Disease-specific incidence, mortality, and prevalence data were collected from the health information databases managed by the Center for Health Development of the Mongolian Ministry of Health. Health information databases manage nationwide health-related data and collect data from all 21 provinces and all nine districts of the Mongolian capital.^14^ Epidemiological data are shown in Supplementary Table S3 and S4. Smoking-related disease-specific cost information was collected in a previous analysis that quantified the inpatient costs for major diseases related to smoking, based on national registry data.^15^ The baseline year for disease incidence and mortality was 2018.

The implementation period of each intervention was set to 3 years. Outcomes were analyzed over a time span of 30 years. Intervention costs were estimated in the local currency Tugrik (1US$ = 2800 Mongolian National Tugrik) at the price level of 2020, and converted to international US$ of 2020 using purchasing power parity rates.^16^ The study adopted a governmental perspective to support policy making concerning tobacco control.

### Interventions

Interventions were selected based on the recent MPOWER country reports that provide information on the level of fulfillment of obligations under the WHO Framework Convention on Tobacco Control.^17^ A comparison of MPOWER measures and current tobacco control policies in Mongolia is shown in Supplementary Table S5. Four tobacco control interventions were selected and independently compared to the business-as-usual (BAU) scenario: School programs, cessation support, mass media campaigns, and tobacco taxes.

### Intervention Effects

The intervention effect size was extracted from the literature, according to the hierarchy of evidence for cost-effectiveness studies evaluations.^18^ Eligible studies in each intervention studied were prioritized in accordance with the following inclusion criteria:

- High-quality study design, such as randomized control trials.
- Studies conducted in Asian countries, that is, intervention effect size of school program extracted from ­studies conducted in China, rather than in European countries.
- Sufficient abstinence time after up to > 12 months.
- Effect of cessation treatment combined with 30-minute consultation.
- Effect of school program combined with 50-minute lesson and self-help manual.
- Smoking prevalence should be comparable to Mongolia, i.e. no influence of bidi smoking.
- Availability of relative risks estimates with 95% confidence intervals (CI).

Table 1 presents the intervention effects used in our model. Studies were prioritized by design type and settings.

**Table 1.** Intervention Effects

Intervention type	Age group, and type of effect	Effect size RR Mean (95% CI)	Reference
Face-to-face interventions			
Smoking cessation*			
Cessation support + NRT	Adults, quit rate	1.47 (1.30 to 1.66)	^19^
School program			
Social influence + curricula	Youth, initiation rate	0.85 (0.72 to 1.0)	^20^
Population-level interventions			
Excise tax**	15–20 years, prevalence	−0.59 (−0.36 to 1.10)	^21,22^ ^23,24^
	21+, prevalence	−0.44 (−0.49 to 0.40)	
Mass media campaign			
Deliver preventive health message and influence health behavior (1-year follow-up)	Youth, initiation rate	0.86 (0.75 to 0.97)	^5,25,26^
	Adults, quit rate	1.496 (1.17 to 1.91)	

^*^Sustained 12 month **Participation price elasticity.

(expressed in thousands).

For smoking cessation support, diverse pharmaceutical therapies have been proposed, with different doses, duration, and schedules. In total, seven studies on patches and nine studies on gum were selected and combined with the help of the MetaXL tool.^27,28^Supplementary Table S6 and Figure S1 present eligible studies including effect size and the pooled effect size for cessation support.

For school programs, the interventions included in previous reviews were carefully checked for feasibility and effectiveness in a Mongolian setting.^20^ This led to the selection of two studies conducted in China, which were then combined on the basis of sufficient follow-up time (>1 year) and a reported intervention effect with a 95% confidence interval (Supplementary Table S7 and Figure S2).

### Participation Rate in Interventions

Depending on the intervention type, whether individual-based or population-based, participation rates varied based on published studies.^5,29^ According to a 2019 national representative survey on the prevalence of noncommunicable disease risk factors in Mongolia (STEPS), approximately 40% of current smokers aged 15 years and older self-reported that they tried to quit smoking in the past 12 months.^30^ In our realistic scenario, at least half of all current smokers who tried to quit smoking would be willing to participate in a cessation support intervention. That is, we applied a participation rate of 20% in our realistic scenario. In our optimistic scenario, the participation rate was set to 40%. For the school program intervention, it was assumed that the program would be implemented country-wide, and hence cover 100% of youths aged 15 to 18.^31,32^ Tobacco taxation and mass media campaigns could by their nature potentially reach 100% of the population.

### Outcome Measures

Health benefits were expressed in DALYs averted and LY gained. Outcome measures were calculated as health benefits for the intervention minus health benefits for the BAU scenario. For LY gained, we used a maximum age of 85 years to remain consistent with the DALY calculations.

### Cost-Effectiveness

To define cost-effectiveness ratios, the sum of additional intervention costs and cost-offsets was divided by the DALYs averted by the intervention. Intervention costs by cost components over 3 years are presented in Supplementary Table S8 and S9. Future costs and effects were discounted at 3%, in line with international guidelines.^33^ Consistent with international guidance, an intervention was considered to be “very cost-effective” if it cost less than the average per capita income to avert one DALY (US$ 4295 in 2020 in Mongolia) and “cost-effective” if it cost less than 3 times the average per capita income (US$ 12 885 in 2019).^34,35^ These thresholds were used to evaluate the various tobacco control interventions.

### Uncertainty Analysis

A range of one-way sensitivity analyses were performed to check the robustness of the cost-effectiveness ratios with regard to variation in model parameter values and model assumptions.

Intervention effects varied by their 95% confidence limits, while the maximum age used in the calculation of health benefits varied from 75 years to 95 years. Intervention costs varied using maximum and minimum estimates for the resources required to implement interventions.

## Results

School programs, tax increases, and mass media campaigns reduce initiation of smoking. All interventions reduce the prevalence of current smoking, with the strongest reduction being observed with increases in tobacco tax (5.1% points), followed by mass media campaigns (1.6% points), school programs (1.3% points), and cessation support interventions (0.6% points) (See Supplementary Table S10 for more details).

Table 2 presents the intervention effects in terms of LYG gained, and DALYs averted, stratified by gender and type of intervention, as net present values. Across all interventions, the most important health gains were obtained in the tax increase scenario: Almost 132–167 thousand DALYs were averted for men and 5–7 thousand for women. The scenarios that led to the lowest number of DALYs averted were the two cessation support scenarios: 12 to 13 thousand DALYs for men and only 0.2 to 2 thousand DALYs for women. School programs and mass media campaigns showed a relatively modest health gain of 27–37 thousand DALYs averted in total, respectively. Regardless of the intervention type, health impacts were much more significant for men than for women.

**Table 2.** Number of Life Year Gained and Number of Disability-Adjusted Life Year Averted by Gender and Intervention Type, Net Present Value Over 30 Years, Maximum Life Expectancy Set to 85 Years, Discounted at 3%

Interventions	Men			Women			Total		
	Mean	Min	Max	Mean	Min	Max	Mean	Min	Max
Number of LY gained									
Taxation	47.0	42.5	53.0	2.1	2.0	2.4	49.1	44.5	55.4
Mass media	2.8	0.6	3.9	3.5	3.5	6.7	6.2	4.1	10.6
School program	6.1	5.7	6.3	4.4	4.4	4.4	10.5	10.1	10.7
Cessation support									
Optimistic scenario	2.7	2.4	2.8	0.1	0.1	0.0	2.8	2.5	2.8
Realistic scenario	2.4	2.2	2.4	0.1	0.1	0.1	2.4	2.2	2.4
Number of DALY averted									
Taxation	146.5	131.7	167.1	6.1	5.4	6.9	152.6	137.1	174.0
Mass media	17.8	6.5	27.2	6.0	5.9	10.2	23.8	12.4	37.4
School program	19.7	18.7	20.5	6.5	6.5	6.5	26.3	25.2	27.0
Cessation support									
Optimistic scenario	13.3	12.2	13.4	0.2	0.2	0.1	13.5	12.3	13.5
Realistic scenario	11.8	11.2	11.9	0.2	0.2	0.2	12.0	11.3	12.0

Intervention effects in terms of LYG and DALY for different assumptions regarding maximum life expectancy (75 years and 95 years) are presented in Supplementary Table S11.

Supplementary Table S12 presents differences in the number of prevalent cases by diagnosis for each of the intervention scenarios compared with BAU. Taking into account former smokers’ influence on disease prevalence, the number of prevented cases varied for each diagnosis and intervention scenario. Lung cancer cases continued to rise during the modelled time span, except for school programmes, due to the persistently high risk of lung cancer for former smokers. Similarly, for all scenarios except school programmes, for all noncommunicable diseases, ischemic heart diseases, stroke, chronic obstructive pulmonary disease, as viewed over the entire time span, the number of prevalent cases increased as a result of longer life expectancy and high risks among former smokers.

Supplementary Table S13 summarizes intervention costs by cost components for tobacco taxation and mass media campaigns. In general, taxation was a more expensive intervention than mass media campaigns, mainly due to the resources required for law development in the first year, and for law reinforcement in later years. Supplementary Table

14 presents cost details for individual-based interventions. The costliest intervention was cessation support, followed by taxation, mass media campaigns, and school programs. School programs were a cost-saving intervention over the 30-year time span, because of a large number of smoking-related diseases thus prevented.

Table 3 and Supplementary Table S15 show costs per LYG and per DALY averted, stratified by type of intervention. School programs were a cost-saving intervention with health benefits, and hence an improvement compared to the BAU scenario. The cost-effectiveness ratio was least favorable for the cessation support scenarios, because of high intervention costs and relatively limited health gains. The cost-effectiveness ratio ranged between US$ 209–293 per LYG, and US$ 62–85 per DALY averted for taxation, and was comparably low for the mass media intervention scenario. Compared to the WHO thresholds, all three population-level interventions could be considered “very cost-effective,” whereas the patient-level interventions were “cost-effective.”

**Table 3.** Cost Per Life Year Gained, Costs Per LY Gained, and Costs Per Disability-Adjusted Life Year Averted, Discounted at 3%, in US$, Price Level 2020, Time Horizon 30 Years. ^*^Each Intervention Scenario Compared to Business as Usual

Intervention	Cost per LY gained			Cost per disability-adjusted life year averted^*^		
	Mean	Min	Max	Mean	Min	Max
Taxation	253	209	293	74	62	85
Mass media	145	dominant**	1163	25	dominant	66
School program	dominant	dominant	dominant	dominant	dominant	dominant
Cessation support						
Optimistic scenario	16 832	16 894	19 019	3477	3475	3786
Realistic scenario	9731	9680	10 466	1961	1958	2062

^*^Assuming a maximum life expectancy of 85 years. **Dominant means cost-saving and health benefits compared to business as usual.

## Discussion

Our analysis suggests that school programs are a cost-saving tobacco control intervention compared to the current practice in Mongolia. All other interventions would be considered very cost-effective since costs per DALY averted were far below US$ 4295 per DALY averted, or the GDP per capita in Mongolia. In terms of health benefits, the most important health gains were observed with tax increases, mass media campaigns, and school programs. Varying intervention effects over their 95% CI had relatively little impact on the incremental cost-effectiveness ratios. The important effect of assuming that all former smokers never smoked indicates that proper information on the prevalence of former smokers is very important, since smoking cessation does not immediately reduce disease and mortality risks. It also explains why school programs, which result in prevention of smoking altogether, and therefore, more individuals who never smoke, were the most cost-effective and even cost-saving policy.

Due to differences among chronic diseases, the number of prevented cases varied per diagnosis. For cancers (lung, oral, and esophageal cancer), an immediate reduction in prevalence numbers occurred among current smokers as a result of population interventions, thanks to the large number of quitters. In the long-term, these quitters would have died in the BAU scenario, given the short survival time for cancers in Mongolia, particularly among men. However, the school program intervention dissuades young people from smoking in the first place, so they are not at risk of smoking-related diseases as they age. Hence the health benefits from this intervention in terms of DALYs averted from cancer increase over time. For chronic diseases (chronic obstructive pulmonary disease, ischemic heart diseases, and Stroke), all interventions were effective over time, yet the most immediate effects were observed in population-based interventions, which increase the quitting rate among adult smokers.

For several reasons, our estimates of cost-effectiveness were conservative. First of all, the model distinguishes between individuals who never smoked and former smokers, which serves to prevent overly optimistic estimates of health benefits. Prevention of smoking, and hence reduced initiation rates among youth leads to the highest health benefits.

Moreover, cost savings from smoking-related diseases were based on estimated inpatient care costs, excluding savings in outpatient and drug costs. Savings from averted productivity losses and health benefits from reduced secondhand smoking were ignored, and would effectively improve the cost-effectiveness of these scenarios. Even given these conservative assumptions, all measures showed a favorable cost-effectiveness when compared to BAU policy in Mongolia.

Other studies on the cost-effectiveness of tobacco control interventions are heterogeneous regarding intervention type, perspective, model structure, and assumptions. For example, Higashi et al. compared the cost-effectiveness of population-based tobacco control interventions such as taxation, mass media campaigns, graphic warning labels on cigarette packs, and smoking bans in Vietnam.^5^ This study found relatively low (highly favorable) incremental cost-effectiveness ratios for interventions that were similar to the ones assessed in the current study. For example, the incremental cost-effectiveness ratios per DALY averted were international dollar (int$) 24.4 for mass media campaigns, and int$ 2.7 for a tax increase from 55% to 85% in Vietnam (PPP adjusted, price level 2016). Another study by the same authors examined the cost-effectiveness of cessation support that combined brief consultations and nicotine replacement therapy (NRT), and found cost-effectiveness ratios from int$20 to int$43 per DALY averted for Vietnam (PPP adjusted, price level 2006). Stephane et al. used an extended cost-effectiveness analysis approach, including effects on productivity and tax revenues to evaluate the consequences of tobacco taxes and smoke-free workplace interventions in China. The study found that a 75% increase in cigarette price would avert about 24 million premature deaths among the male population, and would result in an additional US$ 64 billion tax revenues, while preventing around 9 million poverty cases in China. However, the model structure of this study is static, ignores morbidity gains, and does not discount costs and health outcomes.^36^ In Thailand, the cost-effectiveness of cessation support combined with different NRTs was evaluated from a societal perspective.^37^ The conclusion was that counseling with NRTs was a cost-effective intervention for Thailand.

The strengths of our study are the use of a dynamic multistate model with different smoking categories (never, former, and current smokers). This enables us to present gains in both life years and in quality-adjusted life years or in DALYs averted. Our model’s baseline input parameters are country-specific and taken from country-wide epidemiological registry data on six smoking-related diseases, and from the country’s representative stepwise approach to noncommunicable disease risk factor surveillance (STEP) surveys of smoking prevalence. Also, our cost-effectiveness analysis is based on the government’s perspective. This is in line with our disease-specific costing information, which was collected from public funding resources such as health insurance funding and general budget funding. Unit prices of the NRTs, wage rates, and costs of mass media activities are based on actual prices on the local market.

Although most of our input parameters are country-specific, no local data on the relative risk of smoking, intervention effect sizes, and price elasticity of demand for cigarettes were available. The relative risks used in our model are small compared to the original DYNAMO input data. Our relative risks are taken from studies in Asia, and based on individual-level data originating from more than one million participants in 21 Asian cohort studies.^38^ For example, the relative risk for lung cancer is 3.56 for current smokers, whereas the relative risks used in the original DYNAMO were almost tenfold, based on data from European countries. While the Asian risks seem more appropriate, they do result in lower estimated health benefits of tobacco control policies. In part, these low relative risks reflect the relatively short history of the smoking epidemic in Asia. Thus, over time, the relative risk for many diseases may increase in Mongolia, as individuals who have smoked since their youth grow older. Hence our projection may be overly conservative in this respect.

The intervention effect sizes are taken from literature and might, therefore, be less relevant for the Mongolian setting. We tried to remain on the conservative side to account for this and offer information based on a careful selection of studies.

Cost-effectiveness ratios for both levels for population and individual interventions vary substantially due to the different cost components and long-term health impact, but they are all quite favorable when compared to the GDP per capita thresholds. More intensive tobacco control policy will bring extra benefits not included in these ratios by preventing secondhand smoking, and reducing the costs of tobacco consumption. Regarding the latter, in Mongolia, cessation support medicines are not covered by health insurance, and it may be considerably cheaper to continue smoking than to start using NRT for cessation support for an individual. Most NRTs such as patches and gum are easily available online and at markets without prescription, but the prices are high. On the local market, the most frequently sold brand of cigarettes costs $ 2.77 per pack of 20 in 2018 (PPP adjusted in 2018) in Mongolia, compared to $4 in China, or $ 4.5 in Japan.^1^ A recent STEP survey found that the average smoker spends approximately $ 98 per month on cigarettes, while average monthly income in Mongolia is $690–$970 (PPP adjusted in 2019).^30,39^

In addition, more research is needed to investigate the distributional impact of interventions across a variety of characteristics, including geographic regions and sociodemographics in Mongolia. Moreover, e-cigarettes have become mass-market consumer products in recent years, in particular among young adults. It is therefore worthwhile, in a future evaluation, to consider e-cigarettes and policies addressing their use by children and young adults.

## Conclusion

All tobacco control interventions evaluated score as “very cost-effective” in terms of costs per DALY averted. School programs even appeared to be cost-saving. Population-level interventions such as taxation and mass media campaigns were found to be very cost-effective and brought important health benefits.

## Supplementary Material

A Contributorship Form detailing each author’s specific involvement with this content, as well as any supplementary data, are available online at https://academic.oup.com/ntr.

ntad111_suppl_Supplementary_Material

## Data Availability

All data relevant to the study are included in the article or uploaded as online supplemental information.

## References

[1] WHO report on the global tobacco epidemic. The MPOWER package. Popul Dev Rev. 2008;34(3):581–581.

[2] Beaglehole R , Bonita R, Yach D, Mackay J, Reddy KS. Tobacco-free world 1 A tobacco-free world: a call to action to phase out the sale of tobacco products by 2040. Lancet. 2015;385(9972):1011–1018. 25784348 10.1016/S0140-6736(15)60133-7

[3] National Strategy on Tobacco Control. https://legalinfo.mn/en/edtl/16230948479961 2013. Accessed June 01, 2022.

[4] Hutubessy R , Chisholm D, Edejer TTG. Generalized cost-effectiveness analysis for national-level priority-setting in the health sector. Cost Eff Resour Alloc. 2003;1(1):8. 14687420 10.1186/1478-7547-1-8 PMC320499

[5] Higashi H , Truong KD, Barendregt JJ, et al. Cost effectiveness of tobacco control policies in Vietnam: the case of population-level interventions. Appl Health Econ Health Policy. 2011;9(3):183–196. 21506624 10.2165/11539640-000000000-00000

[6] Drummond M , Barbieri M, Cook J, et al. Transferability of economic evaluations across jurisdictions: ISPOR good research practices task force report. Value Health. 2009;12(4):409–418. 19900249 10.1111/j.1524-4733.2008.00489.x

[7] Feirman SP , Donaldson E, Glasser AM, et al. Mathematical modeling in tobacco control research: initial results from a systematic review. Nicotine Tob Res. 2016;18(3):229–242. 25977409 10.1093/ntr/ntv104

[8] Tuvdendorj A , Du Y, Sidorenkov G, Buskens E, de Bock GH, Feenstra T. Informing policy makers on the efficiency of population level tobacco control interventions in Asia: a systematic review of model-based economic evaluations. J Glob Health. 2020;10(2):020437. 33403106 10.7189/jogh.10.020437 PMC7750019

[9] Bertram MY , Sweeny K, Lauer JA, et al. Investing in non-communicable diseases: an estimation of the return on investment for prevention and treatment services (vol 391, pg 2071, 2018). Lancet. 2018;391(10134):2071–2078. 29627159 10.1016/S0140-6736(18)30665-2

[10] Stefan K , Lhachimi HCB RLM, Wilma J. Nusselder. A Dynamic Model For Health Impact Assessment User Guide 2018. https://www.dynamo-hia.eu/sites/default/files/2018-04/DYNAMO_USERMANUAL_2.0.8_0.pdf.

[11] DALYs GBD , Collaborators H. Global, regional, and national disability-adjusted life-years (DALYs) for 315 diseases and injuries and healthy life expectancy (HALE), 1990-2015: a systematic analysis for the Global Burden of Disease Study 2015. Lancet. 2016;388(10053):1603–1658. 27733283 10.1016/S0140-6736(16)31460-X PMC5388857

[12] Mongolian National Statistic Office. Population Numbers by One Year and Gender. https://www.1212.mn/en/statistic/statcate/573051/table-view/DT_NSO_0300_071V3. Accessed June 01, 2022.

[13] Mongolian Statistical Information Service. https://www.1212.mn/en. Accessed June 01, 2022.

[14] Centre for Health Development in Mongolia. Health Indicator 2018. http://www.hdc.gov.mn/media/uploads/2019-11/2018eng.pdf. Accessed June 01, 2022.

[15] Tuvdendorj A , Dechinkhorloo O, Dorjsuren B, Buskens E, Feenstra T. The costs of inappropriate referral pathways in inpatient care for three major noncommunicable diseases in Mongolia: a national registry-based analysis. BMC Health Serv Res. 2021;21(1):1280. 34838017 10.1186/s12913-021-07281-8 PMC8626993

[16] International Comparison Program, World Bank | World Development Indicators database, World Bank | Eurostat-OECD PPP Programme. https://data.worldbank.org/indicator/NY.GDP.PCAP.PP.CD?locations=MN. Accessed June 01, 2022.

[17] WHO Report on the Global Tobacco Epidemic. https://cdn.who.int/media/docs/default-source/country-profiles/tobacco/who_rgte_2021_mongolia.pdf?sfvrsn=8bdef82a_5&download=true. Accessed June 01, 2022.

[18] Cooper NJ , Sutton AJ, Ades AE, Paisley S, Jones DR; Working Group on the Use of Evidence in Economic Decision Models. Use of evidence in economic decision models: practical issues and methodological challenges. Health Econ. 2007;16(12):1277–1286. 18034447 10.1002/hec.1297

[19] Hartmann-Boyce J , Chepkin SC, Ye W, Bullen C, Lancaster T. Nicotine replacement therapy versus control for smoking cessation. Cochrane Database Syst Rev. 2018;5(5):CD000146. 29852054 10.1002/14651858.CD000146.pub5 PMC6353172

[20] Thomas RE , McLellan J, Perera R. School-based programmes for preventing smoking. Cochrane Db Syst Rev. 2013;8(4):1616–2040. 10.1002/14651858.CD001293.pub3 PMC7028068 23633306

[21] Lim HK , Khang YH. Tobacco price increases in Korea and their impact on socioeconomic inequalities in smoking and subsequent socioeconomic inequalities in mortality: a modelling study. Tob Control. 2021;30(2):160–167. 10.1136/tobaccocontrol-2019-055348 32220983

[22] Kostova D , Tesche J, Perucic AM, et al. Exploring the relationship between cigarette prices and smoking among adults: a cross-country study of low- and middle-income nations. Nicotine Tob Res. 2014;16(Suppl_1):S10–S15. 24343955 10.1093/ntr/ntt170

[23] Nikaj S , Chaloupka FJ. The effect of prices on cigarette use among youths in the global youth tobacco survey. Nicotine Tob Res. 2014;16(Suppl_1):S16–S23. 23709614 10.1093/ntr/ntt019

[24] Cheng KJG , Estrada MAG. Price elasticity of cigarette smoking demand in the Philippines after the 2012 Sin Tax Reform Act. Prev Med. 2020;134(0):106042. 32097751 10.1016/j.ypmed.2020.106042

[25] Bala MM , Strzeszynski L, Topor-Madry R. Mass media interventions for smoking cessation in adults. Cochrane Db Syst Rev. 2017;11(11). 10.1002/14651858.CD004704.pub4 PMC6486126 29159862

[26] Carson KV , Ameer F, Sayehmiri K, et al. Mass media interventions for preventing smoking in young people. Cochrane Db Syst Rev. 2017;6(6). 10.1002/14651858.CD001006.pub3 PMC6481357 28574573

[27] Doi SAR , Barendregt JJ, Khan S, Thalib L, Williams GM. Advances in the meta-analysis of heterogeneous clinical trials I: the inverse variance heterogeneity model. Contemp Clin Trials. 2015;45(1):130–138. 26003435 10.1016/j.cct.2015.05.009

[28] Barendregt JJ , Doi SA, Lee YY, Norman RE, Vos TM. Meta-analysis of prevalence. J Epidemiol Community Health. 2013;67(11):974–978. 23963506 10.1136/jech-2013-203104

[29] Higashi H , Barendregt JJC. Cost-effectiveness of tobacco control policies in Vietnam: the case of personal smoking cessation support. Addiction. 2012;107(3):658–670. 21883602 10.1111/j.1360-0443.2011.03632.x

[30] Third national STEPS Survey on the Prevalence of Noncommunicable Disease and Injury Risk Factors. https://extranet.who.int/ncdsmicrodata/index.php/catalog/836. Accessed June 01, 2022.

[31] Tengs TO , Osgood ND, Chen LL. The cost-effectiveness of intensive national school-based anti-tobacco education: results from the tobacco policy model. Prev Med. 2001;33(6):558–570. 11716651 10.1006/pmed.2001.0922

[32] Holtgrave DR , Wunderink KA, Vallone DM, Healton CG. Cost-utility analysis of the National truth campaign to prevent youth smoking. Am J Prev Med. 2009;36(5):385–388. 19211214 10.1016/j.amepre.2009.01.020

[33] World Health O , Baltussen RMPM, Adam T, et al. Making choices in health: WHO guide to cost-effectiveness analysis/ edited by T. Tan-Torres Edejer... [et al]. Geneva: World Health Organization; 2003.

[34] Murray CJL , Evans DB, Acharya A, Baltussen RMPM. Development of who guidelines on generalized cost-effectiveness analysis. Health Econ. 2000;9(3):235–251. 10790702 10.1002/(sici)1099-1050(200004)9:3<235::aid-hec502>3.0.co;2-o

[35] International Comparison Program, World Bank | World Development Indicators database, World Bank | Eurostat-OECD PPP Programme. https://data.worldbank.org/indicator/. Accessed June 01, 2022.

[36] Verguet S , Tarr G, Gauvreau CL, et al. Distributional benefits of tobacco tax and smoke-free workplaces in China: a modeling study. J Glob Health. 2017;7(2):020701. 29188029 10.7189/jogh.07.020701 PMC5681709

[37] Tosanguan J , Chaiyakunapruk NC. Cost-effectiveness analysis of clinical smoking cessation interventions in Thailand. Addiction. 2016;111(2):340–350. 26360507 10.1111/add.13166

[38] Yang JJ , Yu D, Wen W, et al. Tobacco smoking and mortality in asia: a pooled meta-analysis. JAMA Netw Open. 2019;2(3):e191474. 30924901 10.1001/jamanetworkopen.2019.1474 PMC6450311

[39] National Monthly Salary Schema in Mongolia 2019; Government Order#24:https://legalinfo.mn/mn/detail/14892.

