# Supplementary material for: Cost-Effectiveness of Four Tobacco Control Interventions in Mongolia
Source: Nicotine Tob Res. 2023 Jul 21;25(11):1719–26. doi: 10.1093/ntr/ntad111 (PMC10475601; doi:10.1093/ntr/ntad111)
Supplement: ntad111_suppl_Supplementary_Material [file ntad111_suppl_supplementary_material.docx]

Journal Name: Nicotine Tobacco Research

Title: Cost-effectiveness of four tobacco control interventions in Mongolia

**Appendix**

[Table S 1 Model input parameter 3](#_Toc134717491)

[Table S 2 Observed smoking prevalence by year in Mongolia 4](#_Toc134717492)

[Table S 3 Number of prevalence, incidence and mortality by gender in Mongolia in 2018 5](#_Toc134717493)

[Table S 4 Relative risks of tobacco smoking in Asia ^4 5^ 6](#_Toc134717494)

[Table S 5 Observed smoking Comparison of MPOWER measures and current policy in Mongolia 7](#_Toc134717495)

[Table S 6 Studies selected from cessation support intervention (12m follow up time)^14^ 8](#_Toc134717496)

[Table S 7 Studies selected for school program (12m follow up time) ^15^ 9](#_Toc134717497)

[Table S 8 Intervention costs by cost components over three years 12](#_Toc134717498)

[Table S 9 Cost per participants by type of interventions (In Thousand MNT, year 2020) 12](#_Toc134717499)

[Table S 10 Reduction in smoking prevalence in the third year after intervention 13](#_Toc134717500)

[Table S 11 Number of life years gained and number of DALY averted by intervention type, 14](#_Toc134717501)

[Table S 12 Number of prevented cases, over 30 years 15](#_Toc134717502)

[Table S 13 Intervention costs by resource component over three years (In thousand US$ in 2020) 16](#_Toc134717503)

[Table S 14 Number of participants, price per participant, total intervention costs, costs offset and net costs (in million US$ in 2020) 17](#_Toc134717504)

[Table S 15 Incremental cost-effectiveness for different values of the maximum age. 18](#_Toc134717505)

[Figure S 1 Cessation with more than 12 month follow up time combined with consultation 9](#_Toc93406985)

[Figure S 2 School program with 12 m follow up in China 10](#_Toc93406986)

Table S 1 Model input parameter

| **Input variables in DYNAMO-HIA model** | **Value** | **Sources/reference** |  |
| --- | --- | --- | --- |
| 1. Demographic data | | |  |
| 1. Real population by age and sex specific | 3.3 million | National Statistics Mongolia | ^1^ |
| 1. Overall mortality by age and sex specific | 18.2 thousand | Health info database | ^1^ |
| 1. Fertility forecasting (2015-2045) | 79.6 thousand | National Statistics Mongolia | ^1^ |
| 1. Diseases data: the model included six smoking-related diseases namely oral cancer, esophageal cancer, lung cancer, Chronic Obstructive Pulmonary Disease (COPD), Ischemic Heart Disease (IHD) and stroke. | | |  |
| 1. Incidence by age and sex |  | Health info database | ^2^ |
| 1. Prevalence by age and sex |  | Estimated with DISMOD | ^3^ |
| 1. Excess mortality data by age and sex |  | Estimated with DISMOD |  |
| 1. Relative risk from smoking categories |  | Region specific systematic review | ^4^ ^5^ |
| 1. Disability weights by age and sex |  | Dutch diseases burden study | ^6^ |
| 1. Risk factor data | | |  |
| 1. Smoking categories by age and sex 2. Smoking transition rates by age and sex |  | STEP survey  STEP survey | ^7^ |
| 1. Tobacco-related disease treatment costs (2020, US$) | | |  |
| Oral cancer | 1429 | National tariff list |  |
|  |  |  |  |
| Oespagus cancer | 1429 |  |  |
| Lung cancer | 1802 |  |  |
| COPD | 480 | Author estimated |  |
| Stroke | 525 |  |  |
| Heart disease | 516 |  |  |
| 1. Intervention costs | | | |
| Strategy development |  | State budget regulation | ^8^ |
| Human resources requirements |  | National Wage rate Govt Order #24, 2019 | ^9^ |
| Media advocacy |  | Govt expenses, market data |  |
| Program supplies |  | Govt expenses, market data |  |
| Overhead costs |  | Govt expenses, market data |  |

**Observed smoking prevalence**

Among school children, about 61.3% (65.7% for boys and 51.8% for girls) first tried a cigarette before the age of 14 years. The percentage of students who smoked cigarettes on one or more days during the past 30 days was 9.8% (14.3% for boys and versus 5.7% for girls). Overall, smoking prevalence was higher for urban students than for rural students. It is assumed that over the past 7 years, this prevalence has stayed stable or increased slightly.

For this 2013 round, a multistage, random cluster sampling method was adopted, targeting a set of country representative sites, 65 (32 urban and 33 rural). Randomly selected adults aged 15-64 participated in the survey at each site. Response rates were 98%. Data were available in aggregated form by sex and five-year age categories. When comparing the survey findings from 2013 to 2019, the prevalence of current smoking was reduced for male; from 49% in 2013 to 44% in 2019, whereas the level had stayed stable for women at around 5%.

Table S2 presents observed age-group specific and gender-specific smoking prevalence from periodic Mongolian non-communicable disease STEPwise tobacco surveillance surveys conducted in 2006, 2009 and 2013. ^7^

Table S 2 Observed smoking prevalence by year in Mongolia

| Age group | Male | | | Female | | |  |
| --- | --- | --- | --- | --- | --- | --- | --- |
|  | 2006 | 2009 | 2013 | 2006 | 2009 | 2013 | |
| 15-24 | 23.3 | 35 | 24.9 | 2.1 | 3.9 | 2.3 | |
| 25-34 | 61.7 | 55.5 | 56.7 | 4.7 | 9.1 | 6.7 | |
| 35-44 | 63.1 | 60.3 | 56.6 | 8.7 | 7.2 | 6.2 | |
| 45-54 | 62.2 | 52.3 | 49.2 | 7.7 | 10.9 | 5.2 | |
| 55-69 | 46.6 | 47.5 | 46.9 | 11.6 | 5.4 | 3.9 | |
| **Overall** | **48.4** | **48.0** | **49.1** | **5.5** | **6.8** | **5.3** | |

Model data

### **Smoking prevalence**

Youth smoking prevalence data was collected from the Global School-based Student Health survey, conducted in 2014, the most recent source available. The survey participants were students aged 13-17 years old.^10^ ^11^

Smoking prevalence data for adults were obtained from the “Mongolian STEPwise Approach to chronic disease risk factor surveillance” (STEP) surveys, which provide key information on the major non-communicable disease (NCD) risk factors including smoking prevalence in Mongolia. The most relevant STEP survey which provides information on smoking prevalence in 3 smoking categories, separating never from former smokers, and was conducted in 2013. ^7^ More recent STEP rounds only presented results for smokers versus non-smokers. ^12^ Detailed information was provided in Supplementary Table S2.

Epidemiological data

Disease epidemiology data were collected from the health-info database, which is administered by the National Health Center in Mongolia. The International classification of diseases (ICD-10) were applied, and the following smoking-related diseases were included: ischemic heart diseases (IHD) (I20-I25), stroke (I60-I69; G45), Chronic Obstructive Pulmonary Disease (COPD) (J40-J44), Oral cavity and pharynx cancer (C00-C14), esophageal cancer (C15) and lung cancer (C33-C34). Data were pre-processed to enable parameter estimates for entry into DYNAMO-HIA by using the DISMOD-tool.^3^ Disease-specific incidence, mortality and prevalence data were shown in supplementary Table S3.

The epidemiological causal pathways implies that smoking prevalence is linked through the relative risk (RR) for current smokers versus never smokers and for former smokers versus never smokers. Data on RR were shown in supplementary Table S4.

Table S 3 Number of prevalence, incidence and mortality by gender in Mongolia in 2018

| **List of diseases** | **ICD-10** | **Gender** | **Incidence** | **Mortality** | **Prevalence** |
| --- | --- | --- | --- | --- | --- |
|  |  |  | **(numbers)** | **(numbers)** | **(numbers)** |
| Oral cancer | C00-C14 | male | 65 | 44 | 257 |
|  |  | female | 86 | 36 | 869 |
| Oesophagus (C15) | C15 | male | 177 | 164 | 123 |
|  |  | female | 166 | 152 | 169 |
| Lung cancer (C34) | C34 | male | 319 | 261 | 585 |
|  |  | female | 85 | 58 | 323 |
| COPD (J40-J44) | J40-J44 | male | 3,040 | 85 | 49,850 |
|  |  | female | 5,244 | 55 | 88,420 |
| Stroke (I60-I69, G45) | I60-I69, G45 | male | 2,740 | 935 | 27,771 |
|  |  | female | 4,907 | 1,186 | 55,785 |
| IHD (I20-I25) | I20-I25 | male | 3,872 | 1,483 | 38,275 |
|  |  | female | 5,316 | 1,020 | 74,863 |
| Total | | male | 10,213 | 2,972 | 116,861 |
|  |  | female | 15,804 | 2,507 | 220,429 |

Table S 4 Relative risks of tobacco smoking in Asia ^4 5^

| **Smoking-related diseases** | **Male** | **Female** |
| --- | --- | --- |
| **Relative risk for current smokers versus never smokers** | | |
| Oral (C00-C14) | 2.02(1.89-2.16) | 2.15(1.88-2.47) |
| Oesophagus (C15) | 2.02(1.89-2.16) | 2.15(1.88-2.47) |
| Lung cancer (C34) | 4.80 (3.71-6.19) | 3.53 (2.99-4.16) |
| COPD (J40-J44) | 1.71 (1.51, 1.94) | 2.37 (1.88, 3.00) |
| Stroke (I60-I69, G45) | 1.47 (1.37, 1.58) | 1.73 (1.50, 2.00) |
| IHD (I20-I25) | 1.47 (1.37, 1.58) | 1.73 (1.50, 2.00) |
| Death from all-causes | 1.70 (1.57-1.84) | 1.71 (1.57-1.86) |
| **Relative risk for former smokers versus never smokers** | | |
| Oral cancer (C00-C14) | 1.27(1.16-1.36) | 1.62(1.29-2.04) |
| Esophageal cancer (C15) | 1.27(1.16-1.36) | 1.62(1.29-2.04) |
| Lung cancer (C34) | 4.09 (3.26-5.15) | 3.21 (2.77-3.72) |
| COPD (J40-J44) | 1.87 (1.62, 2.15) | 1.62 (1.08, 2.41) |
| Stroke (I60-I69, G45) | 1.24 (1.14, 1.35) | 1.35 (1.11, 1.65) |
| IHD (I20-I25) | 1.24 (1.14, 1.35) | 1.35 (1.11, 1.65) |
| Death from all-causes | 1.59 (1.50-1.68) | 1.66 (1.55-1.79) |

**Business -as-usual (BAU) scenario:** this indicates the situation where baseline smoking prevalence and baseline smoking behavior will continue with the tobacco measures that are in place and regulated by the current tobacco control law. ^13^

**Smoking cessation support:**

Cessation support was assumed to be available at community and primary health clinics and administered by health care professionals. Based on the available evidence on effectiveness, the intervention includes screening of smoking status upon arrival, and counseling combined with the prescription of the nicotine replacement therapy (NRT), either cytisine, gum or patches ^14^ Accounting for the capacity of routine care and feasibility in practice, the intensity of smoking cessation was defined as the provision of 2 consultations of up to 30 minutes each. Coverage was based on the percentage of adults indicating a wish to quit in the STEP survey. Effectiveness was obtained from a re-analysis of published meta-analyses. ^14^

**School program**

A school program targeted at youth aged between 15 and 18 years old to help young people to not start smoking was evaluated. The scope consists of three lessons, each lasting about 50 minutes. The students are informed about health and mental consequences of smoking, components of smoking and tips that are helpful in resisting cigarettes. The intervention effect was estimated in terms of a relative reduction on youth initiation rates, based on re-analysis of international meta-analyses, selecting studies relevant for the local setting. ^15^ Coverage was assumed to be complete, assuming that the program would be targeted to all students as part of the national school curriculum.

**Mass media**

Mass-media programs were modeled to be broadcasted on TV, social media, and wall posters. Smoking prevalence is expected to decrease from changes in social norm, knowledge, awareness, and belief regarding to smoking. A combined intervention effect in terms of less initiation among young people (aged under 21) and more cessation among adult smokers was modeled. Effect estimates were based on published studies.^16 17^

**Excise tax**

The effect of changes in the price of cigarettes on tobacco consumption was modeled via the price elasticity of demand. Consistent with previous studies, one half of the total price elasticity was assumed to affect smoking participation rates and another half to affect smoking intensity. ^18^ ^19^ We used a smoking participation price elasticity of -0.442 for adult smokers aged over 20 years old from the most recent literature review conducted in Asia. ^20^ For youth, only limited local evidence was available, thus the pooled analysis of the global youth survey was used, which estimated that the smoking participation price elasticity was -0.589 for LMICs. ^21^ Consequently, a 75% price increase may be assumed to lead to a reduction in smoking prevalence of 44% among youths and 33% among adults. Given that most smokers start before the age of 21, we assumed that a reduction in current smoking prevalence due to the intervention would originate from an increase in never smokers among youth, i.e: fewer people starting to smoke, while it would originate from an increase in former smokers among adults, ie: more smokers quitting smoking.

Table S 5 Observed smoking Comparison of MPOWER measures and current policy in Mongolia

| WHO  MPOWER  Measures and scores in 2018 | **M** | **P** | **O** | **W** | **E** | **R** |
| --- | --- | --- | --- | --- | --- | --- |
|  | Monitoring | Smoke-free policies | Cessation support | Warnings/ Mass media | Advertising bans | Raising taxes |
|  | Complete policy | Minimal policy | Moderate policy | Weak policy | Complete policy | Minimal policy |
| Current policy | Periodic representative surveys exist for both adults and youth. | Tobacco control law was adopted in 2012. Smoking is prohibited in all kinds of public transport, public service areas, indoor place and school areas. | NRT* available at some clinics. Varenicline and bupropion are not legally sold, neither covered by health insurance. | Tobacco control law regulates pictorial health warnings on tobacco packages.  Lack of national mass media campaigns designed for youths. ** | Tobacco control law bans advertising, promotion and sponsorship of tobacco products. | The level of excise taxes in the retail price of cigarettes is 38%. Planned to reach at least 60% in 2021. |

NRT*=nicotine replacement therapy

**School-based program will be developed and implemented nation-wide

While category P (smoking-free policy) also scored low, it is hard to develop further scenarios for improvement here, since the tobacco control law prohibits smoking in all public areas including school areas, public transport and indoor/outdoor areas.

Table S 6 Studies selected from cessation support intervention (12m follow up time)^14^

| **Type of NRT** | **Study name** | | **Intervention** | | | | | **Control** | | | |
| --- | --- | --- | --- | --- | --- | --- | --- | --- | --- | --- | --- |
|  |  |  | **N1** | | **Quitters** | | **Non-quitters** | **N2** | **Quitters** | **Non-quitters** | |
| **Gum** | | | | | | | | | | |  |
| 1 | Br thor | | 410 | | 39 | | 371 | 1208 | 111 | 1097 | |
| 2 | Campbell | | 424 | | 13 | | 411 | 412 | 9 | 403 | |
| 3 | Fortman | | 552 | | 110 | | 442 | 522 | 84 | 438 | |
| 4 | Gilbert | | 112 | | 11 | | 101 | 111 | 9 | 102 | |
| 5 | Harackiewicz | | 99 | | 12 | | 87 | 52 | 7 | 45 | |
| 6 | Huges | | 210 | | 23 | | 187 | 105 | 6 | 99 | |
| 7 | Nebot | | 106 | | 5 | | 101 | 319 | 13 | 306 | |
| 8 | Russel | | 729 | | 81 | | 648 | 1377 | 78 | 1299 | |
| 9 | Schneider | | 13 | | 1 | | 12 | 23 | 3 | 20 | |
| **Patch** | | | | | | | | | | |  |
| 10 | Abelin | | 100 | | 17 | | 83 | 99 | 11 | 88 | |
| 11 | Daughan | | 184 | | 25 | | 159 | 185 | 16 | 169 | |
| 12 | Glavas 2003 | | 56 | | 13 | | 43 | 56 | 9 | 47 | |
| 13 | Hays | | 636 | | 62 | | 574 | 322 | 14 | 308 | |
| 14 | Killen | | 212 | | 23 | | 189 | 212 | 21 | 191 | |
| 15 | Otero | | 198 | | 57 | | 141 | 194 | 39 | 155 | |
| 16 | Paolotti | | 60 | | 15 | | 45 | 60 | 4 | 56 | |
| 17 | Sherphof | 251 | | 20 | | 231 | | 267 | 14 | 253 | |


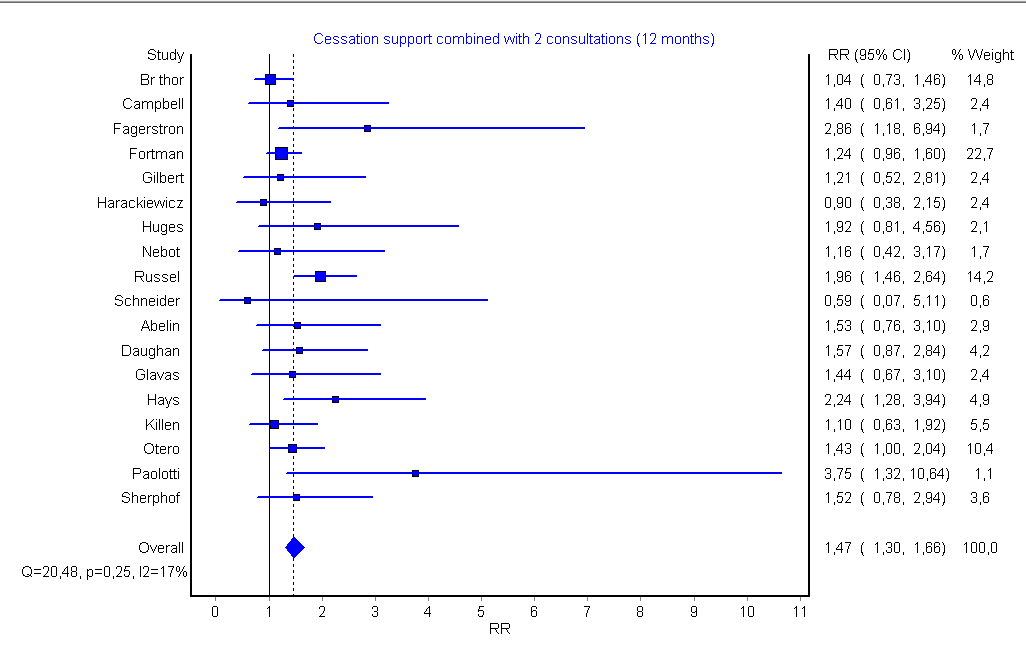
Figure S 1 Cessation with more than 12 months follow up time combined with consultation

Table S 7 Studies selected for school program (12m follow up time) ^15^

| Country | Follow up | Study  name | Active | | | Controls | | |
| --- | --- | --- | --- | --- | --- | --- | --- | --- |
|  |  |  | Total | Quitters | Non-quitters | Total | Quitters | Non-quitters |
| China | 12month | Chou 2006 | 862 | 142 | 720 | 975 | 175 | 800 |
| China | 12month | Wen 2010 | 1162 | 92 | 1070 | 840 | 89 | 751 |


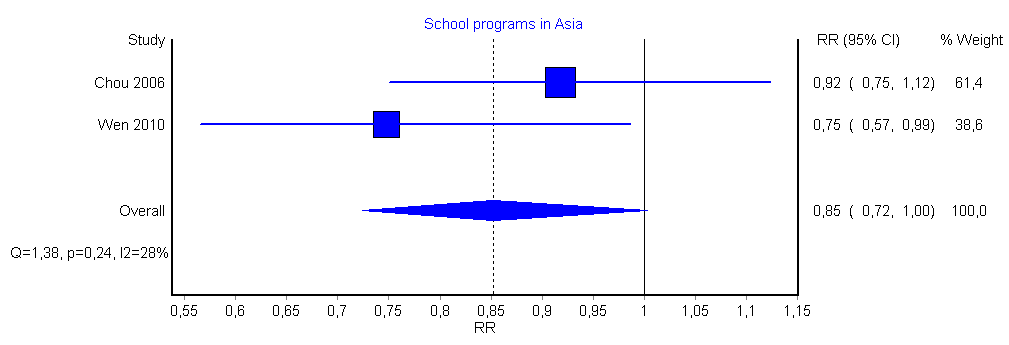


Figure S 2 School program with 12 months follow up in China

### **Costing**

Intervention cost

Depending on the implementation level of each intervention, costs incurred were collected at national, patient and school level. For population-level interventions, the ingredients-based approach was used for population based-interventions.^22^ (Supplementary Table S8) The costIT program developed by the WHO was used to identify resources required in the development and implementation of each intervention.^23^ For individual level interventions, costs were based on resource use as mentioned in the studies underlying the effectiveness estimates, and combined with local costs per unit. (Supplementary Table S9)

Intervention costs were grouped into five main categories: strategy development, human resources, media advocacy, program supplies and overhead costs (rent, utilities, and office supplies). Cost for strategy development and evaluation that include costs for revision of law and guide law implementation, was collected from governmental website and expert consultation. (Supplementary Table S8) The average monthly salary for this sector was based on the national salary schema in 2019. ^25^ Cost for media advocacy includes national and local meetings to disseminate the intervention, educational messages, presentations, TV reports and so on. Actual prices for media advocacy via national TV and daily newspapers were collected from their websites. ^26^ Program supplies and overhead costs (rent, automobile, equipment, utilities) was estimated from the financial report which is publicly available for public authorities in Mongolia. ^27^ The level of overhead cost was estimated as a 40% on human resources costs.

For individual level interventions, costs were based on resource use as mentioned in the studies underlying the effectiveness estimates, and combined with local costs per unit. For cessation support, prices for nicotine replacement therapy (NRT) were based on the national clinical guideline for tobacco us. ^28^ The dosages were listed in this guideline. Prices were based on local market prices, since these NRT (patches, gum and cytisine) are available without prescription. (Supplementary Table S9)

All costs estimates were checked and validated with a national expert to ensure the appropriate values were used in this study. For the school programs, resources use as mentioned in the Chinese study was multiplied with local unit prices. The price per minute of teaching based on the national salary schema in 2019 for the education sector. ^15 25^ Unit prices for printing materials were based on local market prices.

Cost offset

Smoking-related disease costs were acquired from a separate original study, which used data from national inpatient registry records in Mongolia. The study provides mean inpatient costs for patient diagnosed with COPD, IHD and Stroke between 2016 and 2018. For three cancers (lung cancer, oral cancer and esophagus cancer) cost estimates were extracted from the national tariff lists in 2020 for inpatient care services funded by public resources, and consist of health insurance coverage and general government funding.^24^

Table S 8 Intervention costs by cost components over three years

| **Resource component** | **Tobacco taxation** | | | | **Mass media** | | | |
| --- | --- | --- | --- | --- | --- | --- | --- | --- |
|  | **Year 1** | **Year 2** | **Year 3** | **Total costs** | **Year 1** | **Year 2** | **Year 3** | **Total costs** |
| Strategy development and evaluation | 9,500 | - | - | 9,500 | - | - | - | - |
| Human resources requirements | 259,511 | 244,614 | 237,489 | 741,614 | 50,430 | 47,535 | 46,151 | 144,116 |
| Media and advocacy | 82,150 | - | - | 82,150 | 103,960 | 97,992 | 95,138 | 297,090 |
| Programme supplies | 5,100 | - | - | 5,100 | - | - | - | - |
| Rent, utilities, equipment, office supplies | 103,804 | 97,846 | 94,996 | 296,646 | 33,620 | 31,690 | 30,767 | 96,077 |
| **Total costs without discounting** | **460,065** | **342,459** | **332,485** | **1,135,009** | **188,010** | **177,217** | **172,056** | **537,283** |
| **Total costs discounted at 3%** | **446,665** | **322,801** | **304,271** | **1,073,737** | **182,534** | **167,044** | **157,455** | **507,034** |

Table S 9 Cost per participants by type of interventions (In Thousand MNT, year 2020)

| Intervention type | Components | Components | Description | Quantity | Unit price  (min-max) | Total cost per  participant |
| --- | --- | --- | --- | --- | --- | --- |
| Cessation support | Consultations | 30 min x 2 consultations | | 60 | 0.085 (0.075-0.100) | 5.1 (4.5-6.0) |
|  | Patch | 14mg-21mg/24h for  8-10 weeks | 7 patches per pack | 10 | 50 (40-60) | 500 (400-600) |
|  | Gum (2mg) | 10-12 gums/24h x  8-12 weeks | 30 gums per pack | 23 | 50 (40-60) | 1150 (920-1380) |
|  | Cytisine | 1.5mg/ 100 capsules for 20-30 days | 100 capsules per pack | 1 | 100 (85-120) | 100 (85-120) |
| School program | Curricula: | 3 lessons x 50 minute | 25 students per class | 150 | 80 | 0.48 |
|  | Self-help manual |  |  | 1 | 500 | 0.500 |
|  | Anti- smoking poster with 2 messages | 2 message |  | 2 | 200 | 0.300 |

Table S 10 Reduction in smoking prevalence in the third year after intervention

in the total population (compared to a baseline prevalence of 34% for men and 6% for women)

| Interventions | Men | | Women | | Both | |
| --- | --- | --- | --- | --- | --- | --- |
|  | Mean | (Min-Max) | Mean | (Min-Max) | Mean | (Min-Max) |
| **Reduction in the prevalence of current smoking (in percentage points)** | | | | | | |
| Taxation | 10.0 | (8.6-12.2) | 0.4 | (0.3-0.5) | 5.1 | (4.4-6.2) |
| Mass media campaign | 0.9 | (0.2-1.6) | 2.4 | (2.4-3) | 1.6 | (1.2-2.3) |
| School program | 0.15 | (0.1-0.3) | 2.3 | (2.3-2.5) | 1.3 | (1.2-1.4) |
| Cessation support |  |  |  |  |  |  |
| Optimistic scenario | 0.2 | (0.1-0.3) | 1.0 | (1-1) | 0.6 | (0.5-0.6) |
| Realistic scenario | 0.1 | (0.1-0.1) | 0.5 | (0.5-0.5) | 0.3 | (0.3-0.3) |
| **Reduction in current smokers (in thousand)** | | | | | | |
| Taxation | 161.9 | (141.6-195.4) | 8.8 | (8.3-10.4) | 170.7 | (149.9-205.8) |
| Mass media campaign | 13.4 | (0.7-23.8) | 37.7 | (37.8-47.3) | 51.1 | (38.5-71.1) |
| School program | 2.3 | (0-4.6) | 37.1 | (37.2-39.3) | 39.4 | (37.2-43.9) |
| Cessation support |  |  |  |  |  |  |
| Optimistic scenario | 3.0 | (1.9-4) | 15.1 | (15.1-15.1) | 18.1 | (17.1-19.1) |
| Realistic scenario | 1.5 | (1-2) | 7.5 | (7.6-7.5) | 9.1 | (8.5-9.6) |

Table S 11 Number of life years gained and number of DALY averted by intervention type,

gender, and maximum age, net present value over 30 years, discounted at 3%.

| **Intervention type** | **Male** | | | **Female** | | | **Total** | | |
| --- | --- | --- | --- | --- | --- | --- | --- | --- | --- |
|  | mean | Min | Max | mean | Min | Max | mean | Min | Max |
| **LYG** | | | | | | | | | |
| **Age max = 75 years** |  | | | | | | | | |
| Taxation | 46.2 | 41.7 | 52.1 | 1.2 | 1.2 | 1.4 | 47.4 | 42.9 | 53.5 |
| Mass media | 0.9 | 2.5 | 4.2 | 3.5 | 3.5 | 6.8 | 4.5 | 6.0 | 11.0 |
| School program | 5.6 | 5.4 | 5.8 | 4.5 | 4.5 | 4.5 | 10.1 | 9.8 | 10.3 |
| Cessation support |  |  |  |  |  |  |  |  |  |
| Optimistic scenario | 2.7 | 2.4 | 2.7 | 0.1 | 0.1 | 0.0 | 2.7 | 2.4 | 2.7 |
| Realistic scenario | 2.3 | 2.1 | 2.3 | 0.1 | 0.1 | 0.1 | 2.4 | 2.2 | 2.4 |
| **Age max = 95 years** |  |  |  |  |  |  |  |  |  |
| Taxation | 46.6 | 42.1 | 52.5 | 2.4 | 2.3 | 2.7 | 49.0 | 44.4 | 55.3 |
| Mass media | 0.6 | 2.8 | 3.9 | 3.2 | 3.3 | 6.5 | 3.9 | 6.0 | 10.4 |
| School program | 6.1 | 5.8 | 6.3 | 4.2 | 4.3 | 4.2 | 10.4 | 10.0 | 10.5 |
| Cessation support |  |  |  |  |  |  |  |  |  |
| Optimistic scenario | 2.7 | 2.4 | 2.7 | 0.1 | 0.1 | 0.0 | 2.7 | 2.4 | 2.7 |
| Realistic scenario | 2.3 | 2.1 | 2.3 | 0.1 | 0.1 | 0.1 | 2.4 | 2.2 | 2.4 |
| **DALY** | | | | | | | | | |
| **Age max = 75 years** |  |  |  |  |  |  |  |  |  |
| Taxation | 98.7 | 88.6 | 112.9 | 4.3 | 3.8 | 4.9 | 103.0 | 92.4 | 117.8 |
| Mass media | 12.9 | 4.7 | 19.7 | 4.1 | 4.1 | 7.5 | 17.1 | 8.8 | 27.1 |
| School program | 14.0 | 13.4 | 14.6 | 4.6 | 4.5 | 4.6 | 18.6 | 17.9 | 19.2 |
| Cessation support |  |  |  |  |  |  |  |  |  |
| Optimistic scenario | 9.4 | 8.7 | 9.5 | 0.1 | 0.1 | 0.1 | 9.6 | 8.8 | 9.6 |
| Realistic scenario | 8.4 | 8.0 | 8.4 | 0.1 | 0.1 | 0.1 | 8.5 | 8.1 | 8.5 |
| **Age max = 95 years** |  |  |  |  |  |  |  |  |  |
| Taxation | 193.7 | 174.3 | 220.5 | 8.0 | 7.0 | 9.0 | 201.7 | 181.3 | 229.5 |
| Mass media | 22.6 | 8.2 | 34.5 | 7.8 | 7.7 | 12.9 | 30.4 | 15.9 | 47.4 |
| School program | 25.2 | 23.8 | 26.1 | 8.5 | 8.4 | 8.5 | 33.7 | 32.2 | 34.7 |
| Cessation support |  |  |  |  |  |  |  |  |  |
| Optimistic scenario | 17.0 | 15.5 | 17.2 | 0.2 | 0.2 | 0.1 | 17.2 | 15.7 | 17.3 |
| Realistic scenario | 15.1 | 14.3 | 15.2 | 0.2 | 0.2 | 0.2 | 15.4 | 14.5 | 15.4 |

LYG=Life years gains; DALY=disability adjusted life years

Table S 12 Number of prevented cases, over 30 years

(negative numbers present a net increase in the number of cases)

| **Smoking-related diseases** | Taxation | | | Mass media | | | School program | | | Cessation support  (Optimistic scenario) | | | Cessation support  (Realistic scenario) | | |
| --- | --- | --- | --- | --- | --- | --- | --- | --- | --- | --- | --- | --- | --- | --- | --- |
|  | mean | min | max | mean | min | max | mean | min | max | mean | min | max | mean | min | max |
| **Age max = 75 years** | | | | | | | | | | | | | | | |
| Oral cancer | 578 | 525 | 644 | 46 | 28 | 74 | 50 | 49 | 50 | 17 | 15 | 18 | 15 | 13 | 15 |
| Oesophagus cancer | 1282 | 1165 | 1428 | 61 | 28 | 87 | 63 | 59 | 63 | 46 | 38 | 48 | 40 | 34 | 41 |
| Lung cancer | 31 | 25 | 39 | -211 | -237 | -132 | 165 | 154 | 167 | 8 | 8 | 9 | 8 | 8 | 9 |
| COPD | -23060 | -25568 | -21066 | -1102 | -1275 | 1191 | 1368 | 1353 | 1407 | -695 | -707 | -584 | -587 | -592 | -520 |
| IHD | 4593 | 4117 | 5230 | 1214 | 730 | 2150 | 1615 | 1599 | 1647 | 369 | 348 | 379 | 329 | 320 | 331 |
| Stroke | 3900 | 3502 | 4406 | 1058 | 711 | 1754 | 1428 | 1418 | 1450 | 249 | 238 | 261 | 225 | 219 | 226 |
| **Age max= 85 years** | | | | | | | | | | | | | | | |
| Oral cancer | 605 | 549 | 674 | 48 | 29 | 76 | 50 | 49 | 50 | 20 | 16 | 20 | 17 | 15 | 17 |
| Oesophagus cancer | 1474 | 1340 | 1641 | 92 | 55 | 124 | 84 | 75 | 85 | 64 | 52 | 67 | 57 | 47 | 58 |
| Lung cancer | -221 | -242 | -205 | -254 | -275 | -178 | 196 | 180 | 198 | -5 | -6 | -2 | -4 | -5 | -2 |
| COPD | -28320 | -31411 | -25850 | -1612 | -1869 | 507 | 1039 | 1000 | 1060 | -997 | -1031 | -828 | -872 | -887 | -748 |
| IHD | 952 | 803 | 1186 | 792 | 360 | 1650 | 1199 | 1165 | 1239 | 147 | 123 | 159 | 111 | 101 | 142 |
| Stroke | 1141 | 992 | 1343 | 705 | 401 | 1343 | 1090 | 1068 | 1129 | 86 | 64 | 96 | 61 | 54 | 85 |
| **Age max=95 years** | | | | | | | | | | | | | | | |
| Oral cancer | 590 | 535 | 657 | 42 | 24 | 70 | 45 | 45 | 45 | 18 | 15 | 19 | 16 | 14 | 16 |
| Oesophagus cancer | 1439 | 1308 | 1601 | 81 | 45 | 112 | 73 | 66 | 74 | 61 | 50 | 64 | 54 | 45 | 56 |
| Lung cancer | -311 | -343 | -286 | -263 | -281 | -189 | 194 | 178 | 194 | -17 | -19 | -11 | -16 | -16 | -10 |
| COPD | -29502 | -32722 | -26927 | -1893 | -2201 | 149 | 742 | 707 | 835 | -1141 | -1182 | -939 | -1011 | -1028 | -855 |
| IHD | -85 | -141 | 35 | 439 | 49 | 1272 | 876 | 845 | 992 | 21 | -10 | 61 | -12 | -23 | 47 |
| Stroke | 290 | 216 | 400 | 381 | 114 | 997 | 796 | 779 | 906 | -11 | -38 | 21 | -33 | -42 | 12 |

Table S 13 Intervention costs by resource component over three years (In thousand US$ in 2020)

| Resource component | Taxation intervention | | | | | Mass media | | | |
| --- | --- | --- | --- | --- | --- | --- | --- | --- | --- |
|  | Year 1 | Year 2 | Year 3 | Total | Year 1 | | Year 2 | Year 3 | Total |
| Strategy development and evaluation | 3.33 | - | - | 3.33 | - | | - | - | - |
| Human resources requirements | 91.06 | 85.83 | 83.33 | 260.22 | 17.69 | | 16.68 | 16.19 | 50.57 |
| Media and advocacy | 28.82 | - | - | 28.82 | 36.48 | | 34.38 | 33.38 | 104.24 |
| Programme supplies | 1.79 | - | - | 1.79 | - | | - | - | - |
| Rent, utilities, equipment, office supplies | 36.42 | 34.33 | 33.33 | 104.09 | 11.80 | | 11.12 | 10.80 | 33.71 |
| Total intervention costs  (not discounted) | 161.43 | 120.16 | 116.66 | 398.25 | 65.97 | | 62.18 | 60.37 | 188.52 |
| **Total intervention costs**  **(Discounted at 3%)** | **156.72** | **113.26** | **106.76** | **376.75** | **64.05** | | **58.61** | **55.25** | **177.91** |

Table S 14 Number of participants, price per participant, total intervention costs, costs offset and net costs (in million US$ in 2020)

| Interventions | Number of participants | Price per participants | Total intervention costs | Cost-offset | Net costs |
| --- | --- | --- | --- | --- | --- |
|  | (x 10^3^) | in US$ | (x 10^6^) | (x 10^6^) | (x 10^6^) |
| Taxation ^a^ |  |  | 0.4 | 11.0 | 11.4 |
| Mass media^a^ |  |  | 0.2 | 7.7 | 7.9 |
| School programme | 188 | 0.45 | 0.1 | -1.9 | -1.8 |
| Cessation support |  |  |  |  |  |
| Optimistic scenario* | 225 | 206 | 46.5 | 0.8 | 47.3 |
| Realistic scenario** | 113 | 206 | 23.2 | 0.3 | 23.5 |

a Population-level intervention. *In this optimistic scenario, one third of the participants were distributed to each type of NRT. ** In this realistic scenario, half of the participants were distributed to the patch group, and ¼ to the gum group, and another ¼ to the cytisine group.

Table S 15 Incremental cost-effectiveness for different values of the maximum age.

| **Interventions** | Cost per LY gained | | | Cost per DALY averted | | |
| --- | --- | --- | --- | --- | --- | --- |
|  | Mean | Min | Max | Mean | Min | Max |
| **Age max = 75 years** |  |  |  |  |  |  |
| Taxation | 111 | 95 | 126 | 49 | 42 | 54 |
| Mass_media | dominant | dominant | 271 | dominant | dominant | 32 |
| School_program | dominant | dominant | dominant | dominant | dominant | dominant |
| Cessation support |  |  |  |  |  |  |
| Optimistic scenario | 17,124 | 17,060 | 19,330 | 4,854 | 4,851 | 5,253 |
| Realistic scenario | 9,812 | 9,735 | 10,592 | 2,718 | 2,709 | 2,849 |
| **Age max = 95 years** |  |  |  |  |  |  |
| Taxation | 300 | 247 | 348 | 66 | 54 | 76 |
| Mass_media | 303 | dominant | 2,300 | 39 | dominant | 77 |
| School_program | dominant | dominant | dominant | dominant | dominant | dominant |
| Cessation support |  |  |  |  |  |  |
| Optimistic scenario | 17,321 | 17,197 | 19,384 | 2,730 | 2,729 | 2,980 |
| Realistic scenario | 10,003 | 9,976 | 10,703 | 1,546 | 1,545 | 1,627 |

LY=life year; DALY=disability adjusted life year

# **References**

1. National Statistical office of Mongolia, Population annual report. 2019 doi: <http://1212.mn/stat.aspx?LIST_ID=976_L03>

2. Mongolia Cfhdi. Health info databses. 2018 doi: [www.cdh.mohs.mn](file:///C:\Users\ariuntuya\Library\Containers\com.microsoft.Word\Data\Downloads\www.cdh.mohs.mn)

3. Barendregt JJ, Van Oortmarssen GJ, Vos T, et al. A generic model for the assessment of disease epidemiology: the computational basis of DisMod II. *Popul Health Metr* 2003;1(1):4. doi: 10.1186/1478-7954-1-4 [published Online First: 2003/05/30]

4. Zheng W, McLerran DF, Rolland BA, et al. Burden of total and cause-specific mortality related to tobacco smoking among adults aged >/= 45 years in Asia: a pooled analysis of 21 cohorts. *PLoS Med* 2014;11(4):e1001631. doi: 10.1371/journal.pmed.1001631 [published Online First: 2014/04/24]

5. Yang JJ, Yu DX, Wen WQ, et al. Tobacco Smoking and Mortality in Asia A Pooled Meta-analysis. *Jama Netw Open* 2019;2(3) doi: ARTN e191474

10.1001/jamanetworkopen.2019.1474

6. Melse JM, Essink-Bot ML, Kramers PGN, et al. A national burden of disease calculation: Dutch disability-adjusted life-years. *Am J Public Health* 2000;90(8):1241-47. doi: Doi 10.2105/Ajph.90.8.1241

7. WHO. Third national STEPS Survey on the Prevalence of Noncommunicable Disease and Injury Risk Factors-2013.

8. Metrology MAfSa. National Standards 2020(estandard.gov.mn)

9. Mongolia Go. Wage rate in Mongolia. 2019(24) doi: legal.mn

10. WHO. Global School-based Student Health Survey in Mongolia. 2014

11. WHO. WHO report on the global tobacco epidemic, Mongolia. 2019

12. Mongolia PHI. 4th National STEPS Suvery on the prevalence of Noncommunicable disease and injury risk factor. 2018

13. Government of Mongolia. Law of Mongolia on tobacco control. Chapter 1. General

provisions, 2012. Available: <https://www.tobaccocontrollaws.org/files/live/Mongolia/>.

14. Hartmann‐Boyce J, Chepkin SC, Ye W, et al. Nicotine replacement therapy versus control for smoking cessation. *Cochrane Database of Systematic Reviews* 2018(5) doi: 10.1002/14651858.CD000146.pub5

15. Thomas RE, McLellan J, Perera R. School‐based programmes for preventing smoking. *Cochrane Database of Systematic Reviews* 2013(4) doi: 10.1002/14651858.CD001293.pub3

16. Bala MM, Strzeszynski L, Topor‐Madry R. Mass media interventions for smoking cessation in adults. *Cochrane Database of Systematic Reviews* 2017(11) doi: 10.1002/14651858.CD004704.pub4

17. Carson‐Chahhoud KV, Ameer F, Sayehmiri K, et al. Mass media interventions for preventing smoking in young people. *Cochrane Database of Systematic Reviews* 2017(6) doi: 10.1002/14651858.CD001006.pub3

18. World Health Organization. WHO technical manual on tobacco tax administration. World Health Organization, 2010.

19. Chaloupka, Frank J., Kurt Straif, and Maria E. Leon. "Effectiveness of tax and price policies in tobacco control." Tobacco control 20, no. 3 (2011): 235-238.

20. Lim, Hwa-Kyung, and Young-Ho Khang. "Tobacco price increases in Korea and their impact on socioeconomic inequalities in smoking and subsequent socioeconomic inequalities in mortality: a modelling study." Tobacco Control (2020).

21. Kostova D, Tesche J, Perucic AM, Yurekli A, Asma S, GATS Collaborative Group. Exploring the relationship between cigarette prices and smoking among adults: a cross-country study of low-and middle-income nations. nicotine & tobacco research. 2014 Jan 1;16(Suppl_1):S10-5.

22. Organization WH. MAKING CHOICES IN HEALTH: WHO GUIDE TO COST-EFFECTIVENESS ANALYSIS/edited by T. Tan-Torres Edejer. 2003

23. Benjamin Johns* RBaRH. Programme costs in the economic evaluation of health interventions. 2003

24. Communication P. 2020

25. National monthly salary schema in Mongolia 2019;Government Order#24:<http://amc.namem.gov.mn/sanhuu/24.pdf>.

26. Price lists for activities: Mongolian National Broadcasting 2019;2019:<http://www.mnb.mn/s/85?type_slug=i>.

27. Law on transparancy in Mongolia. 2014;<https://www.legalinfo.mn/law/details/10497>

28. Metrology MAfSa. Mental and behavioural disorders due to use of tobacco F17. 2018(<https://estandard.gov.mn/standard/reader/5644>)
